# Controversies in preoperative therapy in esophageal cancer: Current evidence and ongoing research

**DOI:** 10.1002/ags3.12301

**Published:** 2019-11-25

**Authors:** Apurva Ashok, Virendra Tiwari, Sabita Jiwnani, George Karimundackal, C. S. Pramesh

**Affiliations:** ^1^ Division of Thoracic Surgery Department of Surgical Oncology Tata Memorial Hospital Tata Memorial Centre, Homi Bhabha National Institute Mumbai India

**Keywords:** chemoradiotherapy, chemotherapy, esophageal cancer, neoadjuvant

## Abstract

Esophageal cancer incidence is growing worldwide, especially adenocarcinomas in the western world. Outcomes overall are universally poor, with the best survival seen in earlier stages of the disease, where surgery is the mainstay of treatment. Although squamous cell cancers and adenocarcinomas of the esophagus have different etiology, clinical features, biological behavior and prognosis, earlier research studies have frequently combined the two histologies. Several trials in the past three decades have been carried out in the neoadjuvant, adjuvant and perioperative settings in attempts to improve survival further. Most of the initial studies were small and underpowered, and showed no benefit with neoadjuvant or adjuvant treatment over surgery alone. More recent well‐designed trials have now established that the neoadjuvant (in squamous and adenocarcinomas) and the perioperative (in adenocarcinomas) strategies result in superior outcomes compared to surgery alone. However, the optimum neoadjuvant strategy has still not been identified, with both neoadjuvant chemotherapy and chemoradiotherapy (both followed by surgery) showing superior outcomes over surgery alone. Direct comparisons of these two neoadjuvant protocols have not shown a clear benefit of one over the other, although more trials are ongoing and may settle this debate. Future studies using personalized medicine and immunotherapy are required to evaluate their role in the management of esophageal cancers.

## Abstract 2

Several controversies exist about the optimum neoadjuvant treatment strategy for localized esophageal cancer. Both neoadjuvant chemotherapy and chemoradiotherapy (both followed by surgery) show superior outcomes over surgery alone. In this article, we summarize the available evidence, ongoing research and future directions.

## 1 INTRODUCTION

The global incidence of esophageal cancer is on the rise, ranking seventh in terms of incidence and sixth in overall mortality worldwide.^1^ Predominant histological types are adenocarcinoma and squamous cell carcinoma (SCC), which differ in their epidemiology, tumor biology and pathogenesis. Treatment strategies for different histological subtypes should be separate; however, both are traditionally treated primarily with surgical resection.^2^ Low cure rates and poor survival associated with esophageal cancer has brought about a shift in the management strategy from locoregional therapy alone to multimodality regimens. Nevertheless, surgery remains the mainstay of most regimens with addition of chemotherapy or chemoradiotherapy in the treatment of locally advanced disease.^3^

Preoperative therapy (neoadjuvant) appears to offer theoretical advantages over postoperative therapy (adjuvant) to control the micrometastases early. Intact blood supply to the tumor may improve the delivery and effectiveness of chemotherapy and radiotherapy. There is a potential to downstage the tumor and facilitate curative (R0) resection. It may also help to identify tumors with aggressive biological behavior and therefore guide further treatment. However, associated disadvantages of preoperative therapy have to be taken into consideration as it can increase both morbidity and mortality of surgery. There could be technical difficulties of operating in a pretreated field, especially with the addition of radiation, resulting in impaired healing of anastomosis and an increase in postoperative pulmonary complications. Hence, the ideal neoadjuvant treatment should be able to treat micrometastasis, improve survival by preventing local as well as distant failures, and have minimum toxicity and postoperative complications. Although it is possible that chemotherapy and radiation could act synergistically at a locoregional level, the question remains as to whether there is value in combining two local treatments—radiation and surgery. Hence, the optimal treatment regimen for esophageal cancer is still controversial.

## 2 NEOADJUVANT CHEMOTHERAPY

Over the last three decades, extensive research has been done on neoadjuvant treatment for resectable esophageal cancers. Chemotherapy in the preoperative as well as adjuvant setting has been studied. Several randomized trials have compared neoadjuvant chemotherapy (NACT) followed by surgery with surgery alone (Table 1). Two‐drug or three‐drug combinations have been used as first‐line therapy in esophageal cancer. Most of the earlier trials were not adequately powered to definitively answer the question about the value of preoperative chemotherapy.

Among the appropriately powered, large‐scale studies are the US Intergroup trial 113,^4^ which randomized 213 esophageal cancer patients to perioperative chemotherapy (cisplatin + 5‐fluorouracil) and 227 patients to surgery alone. This trial failed to show a significant difference in overall survival (OS) and R0 resection rates (59% vs 63%) between the two groups. Adverse effects of chemotherapy were tolerable and there was no increase in postoperative morbidity or mortality due to the addition of chemotherapy. Both squamous cell carcinoma and adenocarcinoma patients had similar survival curves.

The next large trial was the Medical Research Council (MRC, UK) randomized trial on neoadjuvant chemotherapy in esophageal cancer (OEO2).^5^ It compared 400 patients receiving NACT followed by surgery with 402 patients who underwent surgery alone. Contrary to the Intergroup trial, this study showed a survival benefit with NACT with R0 resection rates (60% vs 54%) and 5‐year overall survival (23% vs 17%) favoring the NACT arm. Postoperative complications were similar in both groups. These treatment results were consistent in adenocarcinoma as well as in SCC patients.

Further trials in the west which investigated the role of NACT were restricted to adenocarcinoma of esophagus and gastroesophageal junction (GEJ). The MAGIC trial^6^ included patients with cancers of the stomach, distal esophagus and GEJ. A total of 503 patients were randomized to perioperative chemotherapy with epirubicin, cisplatin and 5‐fluorouracil (ECF) or surgery alone. Esophageal and GEJ tumors constituted approximately 25% in each arm. Progression‐free survival (PFS) and OS (36% vs 23%) were found to be significantly better in the perioperative chemotherapy arm compared to the surgery arm. Following this study, NACT with ECF became the standard treatment practice for esophageal and GEJ adenocarcinoma in Europe.

The French FFCD study^7^ in 2011 further supported the role of NACT in patients with adenocarcinoma. This trial had a higher percentage of esophageal and GEJ tumours (75% in each arm) and it reported significant survival benefit (both OS and disease‐free survival [DFS]) in the chemotherapy group. Curative resection rates also improved with perioperative chemotherapy. Grades 3 and 4 toxicity was higher in NACT patients receiving cisplatin and 5‐fluorouracil (5‐FU) but postoperative morbidity was similar in both groups.

The Japanese Clinical Oncology Group (JCOG) conducted a trial JCOG 9907^8^ to ascertain the optimal timing of perioperative chemotherapy. A total of 330 esophageal SCC patients were randomized either to postoperative or preoperative chemotherapy with cisplatin and 5‐FU. Analyses showed that the 5‐year survival was better in the preoperative arm (55% vs 43%) without any additional adverse events.

Comparing different chemotherapy regimens, OEO5 and FLOT4 are recent studies of importance. The UK MRC OEO5 trial^9^ compared the conventional cisplatin/5‐FU (CF) regimen with four cycles of epirubicin/cisplatin/capecitabine (ECX) in esophageal adenocarcinoma. R0 resection rates and postoperative complications were similar between the two regimens and there was also no significant difference in median survival (18 months CF vs 21 months ECX), thereby questioning the role of epirubicin in esophageal cancer. The German FLOT4 study^10^ randomized patients with gastric or GEJ adenocarcinoma to the docetaxel, oxaliplatin, 5‐FU with leucovorin (FLOT/ DOF) regimen or to the conventional MAGIC regimen (ECF/ECX). Analyses of 716 patients in this study showed dramatic differences in both PFS as well as 3‐year OS (57% vs 48%) favouring the FLOT arm. With these results, the standard of care for esophageal and GEJ adenocarcinomas seems to be moving towards FLOT rather than the conventional ECF regimen. Most recent trials as well as meta‐analyses^11^, ^12^ clearly show the superiority of neoadjuvant chemotherapy followed by surgery over surgery alone. Importantly, this benefit is seen without an increase in treatment‐related morbidity or mortality.

## 3 NEOADJUVANT CHEMORADIATION

Neoadjuvant chemoradiation (NACRT) has the advantage of combining chemotherapy and radiation prior to surgery, and addressing both locoregional disease as well as micrometastases. Several trials were carried out to evaluate whether neoadjuvant chemoradiation followed by surgery could improve survival over surgery alone (Table 2); most of the earlier studies were small and underpowered to detect a difference. In the Irish trial,^13^ 58 patients were randomized to neoadjuvant chemoradiation with two cycles of 5‐FU and cisplatin with 40 Gy radiation in 15 fractions followed by surgery versus surgery alone. When both groups were compared on an intention‐to‐treat analysis, there was a statistically significant survival advantage in the neoadjuvant group (median survival of 16 months) compared to the upfront surgery group (median survival of 11 months). However, this study was criticized for the small sample size, short median follow up (average 10 months), significant protocol violation (17% in the neoadjuvant group) and poor survival in the surgery arm.^11^

The EORTC trial^14^ also evaluated patients with squamous cell carcinoma and compared neoadjuvant CRT followed by surgery with surgery alone. Here, radiation was delivered in two one‐weekly courses, 2 weeks apart, with five daily fractions of 3.7 Gy each; cisplatin was given before each course of radiation. A total of 282 patients were randomized, 139 to surgery alone and 143 to combined treatment. Complete pathological response was seen in 26% of patients with combined treatment. In this trial, recruitment was stopped earlier than anticipated because of higher mortality in the combined treatment group. After a median follow up of 55.2 months, there was no significant difference in overall survival between the two groups. Esophagus cancer‐related deaths were lower in the neoadjuvant group, although mortality due to other causes was higher. The probable cause of the higher mortality rate was attributed to the higher dose of radiation per fraction.^12^ Drawbacks of this study included the unconventional fractionation, higher dose of radiation per fraction, a 2‐week gap in radiation treatment and the use of cisplatin monotherapy.

The Trans‐Tasman Radiation Oncology Group (TROG) and the Australasian Gastro‐Intestinal Trials Group (AGITG) randomized 256 patients equally to surgery alone (128) or to neoadjuvant chemoradiation followed by surgery (128).^15^ One cycle of cisplatin and 5‐FU was given along with 35 Gy radiation (in 15 days) in the neoadjuvant treatment group. This trial showed no benefit with NACRT in either PFS or OS, although a subset analysis showed superior survival in patients with squamous cell carcinoma. This trial was criticized for the suboptimal dose of radiation (35 Gy) and single cycle of chemotherapy.

The role of NACRT has now been widely accepted globally after the publication of the CROSS trial.^16^ This trial randomized patients in two groups, neoadjuvant chemoradiation followed by surgery and surgery alone. Patients in the neoadjuvant chemoradiation group received weekly carboplatin and paclitaxel for 5 weeks with a radiation dose of 41.4 Gy in 23 fractions. Both groups had 75% adenocarcinoma, 23% squamous cell carcinoma and 2% other histology. Statistically significant improvement in resectability and R0 resections were seen in the neoadjuvant chemoradiation (CRT) group. Median overall survival was 49.4 months in the CRT followed by surgery group and 24 months in the surgery group (*P* = .003). There was no significant difference in postoperative morbidity or mortality in the two groups. Long‐term results of the CROSS trial^17^ confirmed the overall survival advantage with neoadjuvant CRT in all subgroups and also improved DFS, and local and distant recurrence rates. As with neoadjuvant chemotherapy, both meta‐analyses^9^, ^10^ showed superior survival with neoadjuvant chemoradiation followed by surgery compared to surgery alone. In contradistinction to NACT, improved survival with neoadjuvant chemoradiotherapy comes at the cost of increased postoperative morbidity and mortality.^18^, ^19^

## 4 NEOADJUVANT CHEMOTHERAPY VERSUS NEOADJUVANT CHEMORADIOTHERAPY

The important controversy of the optimal neoadjuvant treatment regimen to treat esophageal cancer remains unresolved—there are very few trials comparing NACT with NACRT therapy. The POET trial^20^, ^21^ compared NACT followed by surgery with NACRT followed by surgery; this trial included only locally advanced adenocarcinomas of the esophagogastric junction (Siewert types I and II). Although the study closed early, median OS and PFS showed a statistically insignificant trend favoring the NACRT arm, but the postoperative inhospital mortality was 10.2% in the NACRT arm compared to 3.8% in the NACT arm. Another small Australian trial^22^ compared NACT with NACRT and failed to show a difference in survival; this trial was small and underpowered to show a difference. The recent NeoRes trial^23^ randomized 181 patients to either NACT or NACRT and the study population comprised both adenocarcinoma (73%) and squamous cell carcinoma (27%). Although complete responses (the primary endpoint) and R‐0 resection rates were higher with NACRT, overall survival was identical in the two groups. An updated report with longer follow up^24^ confirmed the lack of benefit in overall survival and there were no differences in recurrence patterns. Notably, although treatment‐related complications were similar in the two groups, postoperative complications were more severe in the chemoradiation group. Overall, there is currently no strong evidence to favor one neoadjuvant strategy over the other; several ongoing trials addressing the problem are likely to answer this question more definitively.

**Table 1:** Summary of neoadjuvant chemotherapy (NACT) trials in esophageal cancer

Trial	Year	No. and treatment	Histology	Location	Endpoints	Results	*P* value
Kelsen et al^4^ (Intergroup 0013)	2007	216: NACT (CF) vs 227: surgery	SCC: 47% ADC: 53%	NA	3 y OS R0 resection	23% vs 26% 63% vs 59%	.74 .5
Allum et al^5^ (OEO2)	2009	400: NACT (CF) vs 402: surgery	SCC: 31% ADC: 67%	M/3: 25% L/3: 64% GEJ: 10%	5 y OS R0 resection	23% vs 17% 60% vs 54%	**.03** **.001**
Cunningham et al^6^ (MAGIC)	2006	250: Periop chemo (ECF) vs 253: surgery	ADC	L/3: 14.5% GEJ: 11.5%	PFS 5 y OS	HR = 0.66 36% vs 23%	**<.001** **.009**
Ychou et al^7^ (FFCD)	2011	113: Periop chemo (CF) vs 111: surgery	ADC	L/3%: 11% GEJ: 64% Gastric: 25%	5 y OS DFS R0 resection	38% vs 24% 34% vs 19% 84% vs 73%	**.02** **.03** **.04**
Ando et al^8^ (JCOG 9907)	2012	166: Postop chemo (CF) vs 164: preop chemo (CF)	SCC	U/3: 9% M/3: 50% L/3: 41%	5 y OS	43% vs 55%	**.04**
Alderson et al^9^ (OEO5)	2017	451: NACT (CF) vs 456: NACT (ECX)	ADC	M/3: 15% L/3 + GEJ: 84%	Toxicity 3 y OS	30% vs 47% 49% vs 42%	**.001** .3
Al‐Batran et al^10^ (FLOT4)	2019	350: NACT (ECF/ECX) vs 356: NACT (FLOT)	ADC	GEJ: 56% Gastric: 44%	5 y OS	36% vs 45%	**.012**

Abbreviations: ADC, adenocarcinoma; CF, cisplatin + 5‐fluorouracil (5‐FU); DOF/FLOT, docetaxel + oxaliplatin + 5‐FU + leucovorin; ECF, epirubicin + cisplatin + 5‐FU; ECX, epirubicin + cisplatin + capecitabine; GEJ, gastroesophageal junction; HR, hazard ratio; L/3, lower third; M/3, middle third; NA, not available; OS, overall survival; PFS, progression‐free survival; SCC, squamous cell carcinoma; U/3, upper third.

Studies with statistically significant results are in bold.

**Table 2:** Summary of neoadjuvant chemoradiation (NACRT) trials in esophageal cancer

Trials	Year	Number	Histology	Location	NACRT regimen	Endpoints	Results
Walsh et al^13^ (Irish)	1996	58: NACRT 55: Surgery	ADC	M/3: 14% L/3: 51% GEJ: 35%	40 Gy/15# + cisplatin + 5‐FU	3 y OS pCR	32% vs 6% 25% vs 0%
Bosset et al^14^ (EORTC)	1997	143: NACRT 139: Surgery	SCC	U/3: 17% M/3: 52% L/3: 31%	37 Gy/10# + cisplatin	5 y OS pCR	26% vs 26% 26% vs 0%
Burmeister et al^15^ (TROG)	2005	128: NACRT 128: Surgery	SCC: 37% ADC: 62%	M/3: 21% L/3: 79%	35 Gy/15# + cisplatin	PFS (SCC) PFS (ADC) OS & PFS	HR: 0.47 (0.25‐0.86) HR: 1.02 (0.72‐1.44) No diff.
van Hagen et al^16^ (CROSS)	2012	180: NACRT 188: Surgery	SCC: 23% ADC: 75%	U/3: 2% M/3: 14% L/3: 58% GEJ: 24%	41.4 Gy/23# + carboplatin/paclitaxel	5 y OS Median OS SCC ADC	47% vs 34% 49 m vs 24 m 82 m vs 21 m 43 m vs 27 m

Abbreviations: ADC, adenocarcinoma; GEJ, gastroesophageal junction; HR, hazard ratio; L/3, lower third; M/3, middle third; OS, overall survival; pCR, pathological complete response; PFS, progression‐free survival; SCC, squamous cell carcinoma; U/3, upper third.

A phase II trial being conducted at the authors’ institute (Tata Memorial Centre), compares NACT with NACRT in squamous esophageal cancer. Similar to the NeoRes trial, the primary endpoint of our trial is response rates, with survival and toxicity being secondary endpoints. The JCOG 1109 NExT trial^25^ is an eagerly awaited study which is a three‐arm trial comparing two‐drug chemotherapy (cisplatin + 5‐FU) with three‐drug chemotherapy (docetaxel + cisplatin + 5‐FU) and neoadjuvant chemoradiation in locally advanced esophageal cancer. The study has overall survival as the primary endpoint with the secondary endpoints being progression‐free survival, R0 resection rates, response rate, pathological complete response rate and adverse events. The ESOPEC trial^26^ is a multicenter phase III German study comparing the efficacy of neoadjuvant chemoradiation (CROSS protocol) versus perioperative chemotherapy (FLOT protocol) in localized esophageal adenocarcinoma; endpoints include survival, treatment‐related morbidity and quality of life. Similar to ESOPEC is the Irish Neo‐AEGIS trial,^27^ comparing the modified MAGIC protocol with the CROSS protocol in adenocarcinoma of esophagus and gastroesophageal junction. A recent modification to this study allows for FLOT to be part of the neoadjuvant chemotherapy arm. These trials should clear the controversy on the optimum neoadjuvant treatment regimen in esophageal cancers. Our calculated guess is that NACRT is likely to be superior to NACT in squamous cell carcinomas and of no additional benefit to NACT in adenocarcinomas.

## 5 TARGETED THERAPY AND IMMUNOTHERAPY

Several years ago, there was a lot of promise and hype about targeted therapy and this has been assessed in a few studies. Panitumumab was evaluated in a German phase II trial (NEOPECX)^28^ and phase III multicentre study (REAL 3),^29^ where patients were randomized to receiving conventional MAGIC (ECX/EOX) with or without panitumumab, three cycles pre‐ and three cycles postoperatively; however, the studies showed no difference in outcomes with the addition of panitumumab. Similarly with bevacizumab, there was a non‐randomized phase II study^30^ and the addition of bevacizumab to cisplatin/5‐FU compared to historical controls showed no difference in outcomes. The MRC phase III trial comparing the addition of bevacizumab to the MAGIC regimen^31^ also showed no evidence for the use of bevacizumab with perioperative chemotherapy. Successes with immunotherapy in several cancers have sparked interest and research in evaluating its role in esophageal cancer. Checkpoint inhibitors are being tested in studies, with observational studies showing remarkable response rates with pembrolizumab^32^ and nivolumab^33^—both drugs have shown response rates superior to chemotherapy alone. However, these were observational studies and need to be validated in well‐conducted randomized trials.

## 6 SUMMARY

Surgery remains the mainstay of treatment for non‐metastatic esophageal cancer. The addition of neoadjuvant treatment is now clearly known to improve outcomes over surgery alone. However, what is unclear is whether neoadjuvant chemoradiation is superior to neoadjuvant chemotherapy alone. Several trials are ongoing which are likely to answer this question. Future trials should also evaluate the potential of immunotherapy to improve outcomes.

## DISCLOSURE

Funding: No funding was received for this study.

Conflicts of Interest: Authors declare no conflicts of interest for this article.
